# Experimental and Computational Characterization of Biological Liquid Crystals: A Review of Single-Molecule Bioassays

**DOI:** 10.3390/ijms10094009

**Published:** 2009-09-10

**Authors:** Kilho Eom, Jaemoon Yang, Jinsung Park, Gwonchan Yoon, Young Soo Sohn, Shinsuk Park, Dae Sung Yoon, Sungsoo Na, Taeyun Kwon

**Affiliations:** 1 Department of Mechanical Engineering, Korea University, Seoul 136-701, Korea; E-Mails:shinedew@korea.ac.kr (J.P.);yo2na@korea.ac.kr (G.Y.);drsspark@korea.ac.kr (S.P.);nass@korea.ac.kr (S.N.); 2 Department of Radiology, College of Medicine, Yonsei University, Seoul 120-752, Korea; E-Mail:177hum@hanmail.net (J.Y.); 3 Department of Biomedical Engineering, Catholic University of Daegu, Kyeongbuk 712-702, Korea; E-Mail:sohnys@cu.ac.kr (Y.S.S.); 4 Department of Biomedical Engineering, Yonsei University, Kangwon-do 220-740, Korea; E-Mail:dsyoon@yonsei.ac.kr (D.S.Y.); 5 Research Institute of Engineering and Technology, Korea University, Seoul 136-701, Korea

**Keywords:** single-molecule experiments, atomic force microscope (AFM), coarse-grained molecular dynamics simulation, coarse-grained model, *in vitro* molecular recognitions

## Abstract

Quantitative understanding of the mechanical behavior of biological liquid crystals such as proteins is essential for gaining insight into their biological functions, since some proteins perform notable mechanical functions. Recently, single-molecule experiments have allowed not only the quantitative characterization of the mechanical behavior of proteins such as protein unfolding mechanics, but also the exploration of the free energy landscape for protein folding. In this work, we have reviewed the current state-of-art in single-molecule bioassays that enable quantitative studies on protein unfolding mechanics and/or various molecular interactions. Specifically, single-molecule pulling experiments based on atomic force microscopy (AFM) have been overviewed. In addition, the computational simulations on single-molecule pulling experiments have been reviewed. We have also reviewed the AFM cantilever-based bioassay that provides insight into various molecular interactions. Our review highlights the AFM-based single-molecule bioassay for quantitative characterization of biological liquid crystals such as proteins.

## 1. Introduction

Biological liquid crystals such as proteins perform excellent mechanical functions. For instance, spider silk has been recently found to exhibit excellent mechanical properties such as high extensibility (>100%), as well as high yield stress (comparable to that of high-tensile steel) [1,2]. The muscle protein titin performs mechanical functions such as muscle contraction and relaxation through unfolding and/or refolding of hydrogen bonds [3–6]. This indicates that a quantitative understanding of the mechanical responses of protein molecules is essential for gaining insight into their biological functions.

Mechanical characterization of proteins at single-molecule level has been facilitated by micro- and/or nano-technology techniques that have allowed the development of single-molecule force spectroscopy based on atomic force microscopy (AFM) [3–5], laser tweezer (LT) [6–8], and/or single-molecule imaging technique based on fluorescence resonance energy transfer (FRET) [9,10]. Quantitative understanding of the mechanical response of a biomolecule was first provided by Bustamante and coworkers [11], who reported the entropic elasticity of DNA molecule using a LT bioassay. Since then, the LT bioassay has been broadly employed for quantitative studies on mechanics of DNA molecules relevant to their biological functions [6]. Marszalek *et al*. [12,13] first reported the mechanical response of muscle protein titin using an AFM bioassay. In their experiment [12,13], it is shown that the unique feature in the force-displacement curve (*i.e.,* saw tooth-like force curve) can be ascribed to the unfolding of folded domains. Since this pioneering work the AFM bioassay has been extensively considered for quantitative understanding of protein unfolding mechanisms and/or bond rupture mechanisms [14,15]. Despite allowing a quantitative characterization of the mechanical behavior of biomolecules, the AFM bioassay (or LT bioassay) may not provide the details of the mechanical response such as protein unfolding pathways.

As stated above, single-molecule experiments exhibit limitations in gaining insight into detailed protein unfolding mechanisms. Computational simulations such as molecular dynamics (MD) simulations have enabled the description of some detailed mechanisms of protein unfolding mechanics such as unfolding pathways [16–19]. Nevertheless, MD simulations are sometimes computationally unfavorable for large protein complexes due to the limited simulation time-scales, much smaller than those relevant to single-molecule experiments [20]. This implies that current MD simulation provides only a qualitative understanding of protein unfolding mechanisms. In order to overcome such limitations, coarse-grained MD simulations [21–24] have attracted much attention. Specifically, unlike all-atom MD simulations, coarse-grained MD simulations are implemented by reduction of degrees of freedom as well as simplification of the potential field [25]. Such a coarse-grained MD simulation has allowed the quantitative insight into protein unfolding mechanism on a time-scale relevant to single-molecule experiments. Moreover, in recent decades, the AFM bioassay has enabled not only single-molecule pulling experiments, but also an understanding of various molecular interactions relevant to early diagnosis of specific diseases [5]. Specifically, the cantilever surface is chemically modified in order to functionalize the specific receptor molecules that are capable of capturing the specific target molecules. The fundamental feature in such a cantilever bioassay is the direct transduction of molecular interactions on the cantilever surface into a mechanical response change of the cantilever such as a bending deflection change [26] and/or a resonant frequency shift [27]. Such a bioassay has been ascribed to Gerber and coworkers [28], who took into account a cantilever bioassay for gaining insight into intermolecular interactions between alkanethiol chemical groups. Since then, numerous studies on molecular interactions using cantilever assays have been widely performed.

In this work, we have extensively reviewed the current state-of-art in quantitative characterization of biological liquid crystals based on single-molecule experiments and/or computational simulations. Single-molecule pulling experiments based on AFM- or LT-bioassay have been briefly overviewed. Computational simulations such as coarse-grained MD simulation have been reviewed in detail. In addition, we have also taken into account the current state-of-art in cantilever-based bioassays for quantitative understanding of molecular interactions. Our study sheds light on AFM-based single-molecule experiments and/or single-molecule pulling simulation for quantitative characterization of biological liquid crystal properties such as protein unfolding mechanics and/or protein-protein interactions relevant to early diagnosis of specific diseases.

## 2. Single-Molecule Pulling Experiments

**Figure 1.**
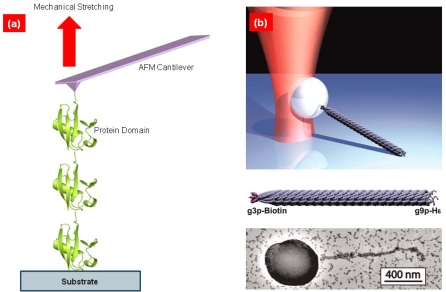
(a) Schematic illustration of atomic-force-microscopy (AFM)-based single-molecule pulling experiments of biological molecule: One end of biomolecule is attached to the substrate, while the other end attached to AFM cantilever tip is stretched by manipulation of AFM. (b) (Top) Schematic of laser tweezer (LT)-based single-molecule stretching of biomolecule: One end of biomolecule is attached to the substrate whereas the other end attached to nanoparticle that can be trapped by LT is stretched by control of LT. (Middle) Schematic illustration of biomolecular structure (Bottom) Microscope image of biomolecule attached to nanoparticle that could be trapped by LT. Figures are adopted from Ref. [29].

**Figure 2.**
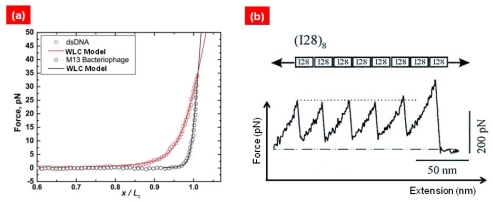
(a) Typical force-extension of biomolecule such as double-stranded DNA or bacteriophage obtained from LT bioassay. The force-extension curves of dsDNA and/or bacteriophage are well fitted to worm-like-chain (WLC) model. Figures are adopted from Ref. [29]. (b) Force-extension curve of muscle protein titin immunoglobulin (Ig) 28 domains obtained from AFM bioassay. The force peak in force-extension curve corresponds to the unfolding of a single Ig 28 domain. Figures are adopted from Ref. [41].

In 1994, Bustamante and coworkers [11] had first suggested the LT bioassay for quantitative understanding of the entropic elasticity of DNA molecules. In their experiment [11], the ends of a DNA molecule are attached to beads, one of which can be trapped by LT while the other is stretched by a micropipette. Such an LT bioassay provides the relation of force-extension of biomolecules [29] (see Figure 1). Based on such a force-extension curve, Bustamante and coworkers first showed that elastic response of DNA molecule is well fitted to a worm-like chain (WLC) model [30,31] (see Figure 2), which provides the force-extension relation given by

(1) $$F=\frac{k_{B}T}{\xi_{p}}\left[\frac{1}{4}{\left(1-\frac{x}{L_{0}}\right)}^{-2}\;-\frac{1}{4}+\frac{x}{L_{0}}\right]$$

where *F* is the force applied to a molecule, *x* is the extension of a molecule, *k_B_* is the Boltzmann constant, *T* is the absolute temperature, *ξ_p_* is the persistence length (related to bending rigidity) of a molecule, and *L*_0_ is the contour length of a molecule. So far, the LT bioassay has been widely utilized for characterization of elastic responses of DNA molecules [6,32,33], including even short DNA molecules [34] and/or RNA molecules [8,35–37].

Except for LT bioassays, AFM-based single-molecule pulling experiments have been broadly employed for mechanical characterization of not only DNA/RNA molecules [38,39] but also protein molecules [3–5] and/or bond rupture mechanisms [14,15] (see Figure 1). Unlike DNA molecules, single-molecule pulling experiments of protein molecules provides the saw tooth-like force-extension curve which arises from the unfolding of folded domains (see Figure 2). Nonetheless, before folded domains are unfolded, the mechanical response of protein is well depicted by the WLC model. This led Rief and coworkers [40] to provide a simple model for quantitative characterization of protein unfolding experiments by using a WLC model (for a domain) with bond rupture model (for unfolding). More remarkably, the force-extension curve also enables the characterization of molecular structures such as misfolded domains [41]. This indicates that, unlike entropic elasticity in polymer physics, the mechanical response of protein molecule is governed by the native topology (of folded domains) [42]. Recently, Rief and coworkers [43–45] have remarkably shown the anisotropic mechanical response of green fluorescence protein (GFP) by considering the different pulling geometries. This is ascribed to geometry of hydrogen bonds responsible for the protein unfolding mechanism. In the similar manner, they have also showed that telethonin connecting Z1–Z2 domains in muscle protein plays a vital role on resistance to mechanical force acting on specific directions [46]. Here, it should be noticed that pulling direction plays a significant role on the mechanical (entropic elastic) response of biomolecules. For instance, recent studies [47,48] show that, for an AFM single-molecule pulling experiment, the pulling of DNA molecules with an angle with respect to contour length affects the force-extension curve of DNA molecules. This indicates that force-extension curve obtained from AFM single-molecule pulling experiments has to be carefully considered for mechanical characterization of DNA/RNA molecules. This feature may be also found in case of stretching of protein molecules using AFM force spectroscopy. To the best of our knowledge, such an issue has been rarely taken into account, except for a recent numerical study [49] showing that stretching of protein-like folded structures with a specific angle with respect to contour length has an effect on the force-extension curve. This implies that protein unfolding mechanics may be dependent on the angle between the direction on which the force is acting on and the contour length. It is implied that there might be an error induced from AFM pulling process when a biomolecule is stretched in a different direction from that of contour length [48]. Moreover, it should be recognized that the loading rate accessible with LT bioassays (in the range of ~1 pN/s [50]) is much less than that available for AFM bioassays (in the range of 10^5^~10^6^ pN/s [44]). This suggests that LT bioassays are able to provide the quasi-equilibrium stretching of biomolecule, which cannot be achieved with an AFM bioassay. In addition, the force resolution for LT bioassays is in the range of ~10 pN [50], while the force resolution for AFM bioassays is >50 pN [38,44]. Until recently, LT bioassays have been mostly employed for stretching of DNA/RNA molecules, while protein unfolding mechanics have been intensively studied with AFM force spectroscopy.

Single-molecule pulling experiments (using LT or AFM bioassays) have allowed the verification of theory in statistical mechanics. Bustamante and coworkers [50] have notably verified Jarzynski’s theorem [51] based on LT bioassay-based single-molecule pulling experiments of RNA hairpin. Specifically, Jarzynski’s theorem [51] provides the free energy difference between two equilibrium states estimated from non-equilibrium experimental process [50,52]

(2) $$\langle \text{exp}\left(-\frac{W}{k_{B}T}\right)\rangle =\text{exp}\;\left(-\frac{\text{Δ}G}{k_{B}T}\right)$$

**Figure 3.**
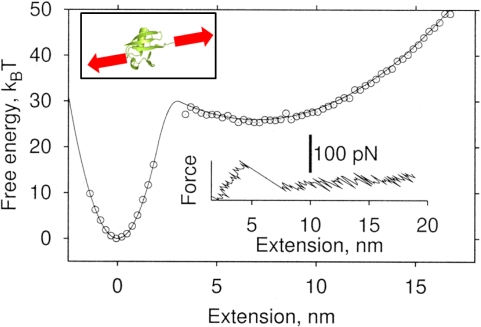
Free energy landscape, for protein, reconstructed from single-molecule pulling simulation with Jarzynski’s theorem: Inset above shows the schematics of stretching of a single protein domain by mechanical force. Inset below provides the force-extension curve obtained from simulation. Figures are adopted from Ref. [52].

Here, *W* is the work done during the non-equilibrium process, Δ*G* is the free energy difference between two equilibrium states, and <*Z*> is the ensemble average of quantity *Z* (see Figure 3). In the work by Bustamante and coworkers [50], the unfolding and/or the refolding of RNA hairpin was taken into account for measurement of free energy differences between an unfolded state and the folded state based on fast pulling experiment (non-equilibrium process) with Jarzynski’s theorem and/or slow pulling experiments (equivalent to quasi-equilibrium process). In a similar spirit, the free energy landscape related to protein unfolding has been quantitatively characterized using Jarzynski’s theorem with the force-extension curve for protein unfolding from AFM single-molecule pulling experiments [52,53] (see Figure 3). Moreover, Bustamante and coworkers [54] have verified Crooks’ theorem that provides the free energy difference from the probability distributions of the work done during non-equilibrium process

(3) $$\frac{P_{U}(W)}{P_{R}(-W)}=\text{exp}\left(\frac{W-\text{Δ}G}{k_{B}T}\right)$$

where *P_U_*(*W*) is the probability distribution of the work done during unfolding process, and *P_R_*(–*W*) the probability distribution of the work done during refolding process.

**Figure 4.**
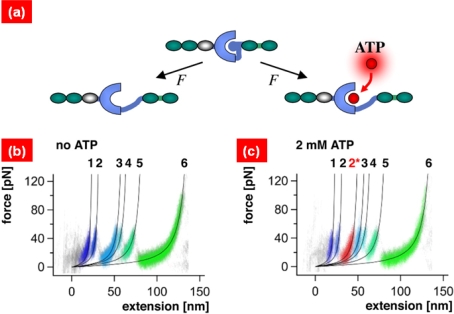
(a) Schematic illustration of stretching of titin kinase: Stretching of titin kinase enables the opening of binding site onto which ATP molecule can be bound. (b) Force-extension curve of titin kinase obtained from AFM bioassay. (c) Force-extension curve of titin kinase in the presence of ATP molecule. The asterisk indicates the unique feature in force-extension curve due to ATP binding. This implies the enzymatic activity of titin kinase by mechanical force. Figures are adopted from Ref. [56].

Recently, single-molecule pulling experiments have allowed one to gain insight into the role of mechanical force on the binding affinity. For instance, Fernandez and coworkers [55] have considered LT bioassays in order to find the role of mechanical force on the binding affinity for talin rod molecules. In their work [55], it is shown that stretching a talin rod molecule increases the binding affinity of ths talin rod molecule to vinculin. In a similar spirit, Gaub and coworkers [56] have examined the AFM bioassay-pulling experiment the effect of titin kinase upon ATP binding (see Figure 4). It is found that ATP binding onto titin kinase affects the mechanical response of protein unfolding mechanism, indicating that titin kinase acts as a biological force sensor.

Moreover, single-molecule pulling experiments have provided insights into the energy landscape related to protein folding and/or bond ruptures. Specifically, the unfolding force (and/or probability distribution of unfolding forces) with respect to pulling rate is related to the free energy barrier and/or barrier width relevant to protein folding. Such a relationship has been attributed to Bell [57], who first provided the relationship between kinetic rate for bond rupture and mechanical force inducing bond rupture. The theoretical backgrounds on bond rupture mechanism are described as below.

## 3. Bond Rupture Models: From Bell’s Theory to Dudko-Hummer-Szabo (DHS) Theory

The role of mechanical force on the bond rupture mechanism was first observed in cell adhesion problems. Specifically, the detachment of cell on the substrate via bond rupture driven by mechanical force was theoretically considered by Bell [57]. In Bell’s model [57], the relationship between kinetic rate for bond rupture and force was empirically represented in the form of an Arrhenius equation [58]

(4) $$k(f)=\chi_{0}\;\text{exp}\left(\frac{\text{Δ}G_{b}-f\;\text{Δ}x_{b}}{k_{B}T}\right)$$

where *k*(*f*) is the kinetic rate for bond rupture in the presence of mechanical force *f*, *χ*_0_ is the natural frequency of a bond, Δ*G_b_* is the energy barrier, and Δ*x_b_* is the energy barrier width. When a force is applied to a chemical bond, the free energy landscape for a bond is tilted by mechanical force. Here, in Bell’s model, the energy barrier width Δ*x_b_* is independent of mechanical force, which is only relevant to small force regime. Consequently, Bell’s model is only appropriate for small force applied to a chemical bond.

Evans and Ritchie [14,59] have revisited Kramers’ theory [60] in order to describe the bond rupture mechanism upon mechanical force. Kramers’ theory [60] is extracted from the Smolouchowski equation [61–63] represented in the form

(5) $$\frac{\partial }{\partial t}\rho (x,t;x_{0})=\frac{\partial }{\partial x}D\left[k_{B}T\frac{\partial }{\partial x}\rho (x,t;x_{0})+\rho (x,t;x_{0})\frac{\partial V}{\partial x}\right]\equiv ℘\;\rho (x,t;x_{0})$$

Here, *ρ*(*x*, *t; x*_0_) is the probability density for an intact contact at position *x* and time *t* under the initial position *x*_0_, *D* is the diffusion coefficient, *V* is the effective potential relevant to bond rupture by mechanical force, and ℘ is the differential operator. The probability for an intact contact at time *t* is easily given by *S* (*x*_0_,*t*) = ∫ *dx* *ρ*(*x, t; x*_0_) = ∫ *dx* exp[*t*℘] · *δ*(*x* – *x*_0_), where δ(*z*) is the Dirac delta function.

The unfolding time for a bond rupture by mechanical force is given by

(6) $$\tau (x)=\int_{x}^{b}dy\;\text{exp}\;\left[\frac{V(y)}{k_{B}T}\right]\frac{1}{D}\int_{a}^{y}dz\;\text{exp}\;\left[-\frac{V(z)}{k_{B}T}\right]$$

Based on *V*(*x*) = *V*_0_(*x*) – *fx*, where *V*_0_(*x*) is the potential energy for a bond without any force *f*, and Taylor series expansion of Equatation (6), the kinetic rate for a bond rupture is

(7) $$k(f)\equiv \frac{1}{\tau (f)}\approx \frac{D\omega_{b}(f)\omega_{ts}(f)}{2\pi k_{B}T}\text{exp}\;\left[\frac{\text{Δ}V_{0}(f)-f\;\text{Δ}x_{b}(f)}{k_{B}T}\right]$$

where *ω_b_* and *ω_ts_* are natural frequencies at equilibrium state for bond formation and transition state at which unfolding process is initiated, Δ*V*_0_(*f*) is the force dependent energy barrier, and Δ*x_b_*(*f*) is the force dependent energy barrier width. Dudko *et al*. [64] showed that, for fast pulling rate, the bond rupture behavior is quite different from that suggested by Bell’s model using escape field theory [65].

Dudko, Hummer, and Szabo [66] have taken into account Kramers’ theory [60] given by Equation (10) with a presumed potential energy profile in order to find the kinetic rate for protein unfolding mechanics. They have considered a linear-cubic potential and cusp potential for extraction of a kinetic rate. The kinetic rate suggested by Dudko *et al*. [66] referred to as DHS model is represented in the form of

(8) $$k(f)=k_{0}\;{\left(1-\frac{v\;fx^{†}}{\text{Δ}G^{†}}\right)}^{1/v-1}\;\text{exp}\left[\text{Δ}G^{†}\;\left\{1-{\left(1-\frac{v\;Fx^{†}}{\text{Δ}G^{†}}\right)}^{1/v}\right\}\right]$$

**Figure 5.**
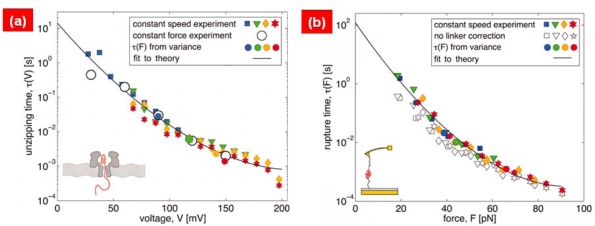
(a) Kinetic rate for RNA hairpin unzipping by nanopore-based bioassay is compared with Dudko-Hummer-Szabo (DHS) model. (b) Comparison of kinetic rate for mechanical unfolding of protein with DHS model. It is shown that kinetic rate for mechanical unfolding and/or unzipping of folded domain can be well described by DHS model. Figures are adopted from Ref. [67].

Here, it is assumed that *x* = 0 corresponds to the equilibrium state for a bond, and that *x* = *x*^†^ corresponds to the transition state related to bond rupture. A parameter *ν* represents the shape of free energy landscape, *i.e.,* *ν* = 2/3 for linear-cubic potential, *ν* = 1/2 for cusp potential, and *ν* = 1 corresponds to Bell’s model. The kinetics of protein unfolding based on AFM single-molecule pulling experiments and/or nanopore translocation experiments have been well described by DHS model [67] (see Figure 5).

## 4. Computational Simulations: Coarse-Grained Molecular Dynamics (MD) Simulations

As stated above, single-molecule pulling experiments are not sufficient to provide insight into protein unfolding mechanisms. For instance, single-molecule pulling experiments cannot suggest the details of mechanical unfolding pathways. In recent decades, molecular simulations such as molecular dynamics (MD) simulations [68] have allowed a detailed description of protein dynamics such as conformational transitions [69,70] and/or protein unfolding mechanics [19].

The fundamental task of a MD simulation is to solve the equation of motion for all atoms prescribed by an empirical, complicated potential field. Even though MD simulations provide the details of protein dynamics, the time-scale available for MD simulation is much less than that relevant to single-molecule experiments [20]. This leads to gap between experiments and MD simulation such that the pulling rate typically used in MD simulation is much larger than that relevant to AFM bioassay-based single-molecule pulling experiment by factor of ~10^5^. In order to resolve such a gap between simulation and experiment, coarse-grained MD simulation [71] has been introduced by considering only the alpha carbon atoms with a simplified potential field. One of popular coarse-grained MD simulations is the Gō-like model [21,22,72,73], which was first suggested by Gō and coworkers [74] for studying protein folding processes based on alpha carbon atoms (with bond formation) in the backbone chain. In this article, the Gō-like model is only limited to a review of simulation of protein unfolding mechanics, although a Gō-like model also allows the simulation of protein dynamics (conformational dynamics) [75–78] as well as protein folding [74]. The Gō model assumes that alpha carbon atoms are prescribed by the potential field given by

(9) $$V=\sum_{i}\left[\frac{k_{1}}{2}{\left(d_{i}-d_{i}^{0}\right)}^{2}+\frac{k_{2}}{4}{\left(d_{i}-d_{i}^{0}\right)}^{4}\right]+\sum_{j\neq i}4{ɛ}_{0}\left[{\left(\frac{\sigma }{r_{ij}}\right)}^{6}-{\left(\frac{\sigma }{r_{ij}}\right)}^{12}\right]$$

Here, *d_i_* is the bond vector defined as *d_i_* = |**r***_i_* – **r***_i_*_+1_| with **r***_i_* being the position vector of *i*-th alpha carbon atom, *r_ij_* is the distance between two alpha carbon atoms *i* and *j*, *i.e., r_ij_* = |**r***_i_* – **r***_j_*|, *k*_1_ and *k*_2_ are force constants for harmonic and quartic potentials for covalent bond stretching, *ɛ*_0_ is the energy parameter for native contact, *σ* is the length scale relevant to native contact, and superscript 0 indicates the equilibrium state. In the similar spirit, self-organized polymer (SOP) model has been introduced by considering the effective potential prescribed to alpha carbon atoms (for details, see Ref. [45,79–81]).

Based on Gō potential, the unfolding pathway of protein from coarse-grained MD simulation is obtained from Langevin equation given as [82]

(10) $$m{\ddot{\text{r}}}_{i}(t)+\sum_{j\neq i}^{N}{\text{D}}_{ij}\cdot {\text{r}}_{j}(t)+\frac{\partial }{\partial {\text{r}}_{i}}\tilde{V}({\text{r}}_{1},\cdots ,{\text{r}}_{N})={\text{F}}_{i}(t)$$

where *m* is the molecular weight of carbon atom, **D***_ij_* is the 3×3 matrix to represent the hydrodynamic i nteractions between alpha carbon atoms *i* and *j* [61,82], and dot indicates the differentiation with respe ct to time *t*. For computational efficiency, **D***_ij_* is simplified such that **D***_ij_* = *γδ_ij_* [21,22,72,73], where *δ_ij_* is the Kronecker delta, and **F***_i_*(*t*) is the random force due to thermal fluctuation, *i.e.,* **F***_i_*(*t*)·**F***_j_*(0) = 2**D***_ij_δ*(*t*) [61,82], where *δ*(*t*) is the Dirac delta function. Herein, it should be noted that solvent is considered as continuum dictated by hydrodynamic tensor **D***_ij_*, similar to implicit solvent in MD simulation. Here, *Ṽ* is the effective potential including the potential given by Equation (4) and the work done by AFM pulling process. For stretching a biomolecule with a constant pulling speed *u*, the effective potential is given by

(11) $$\tilde{V}=V+\frac{k_{c}}{2}{\left(R-ut\right)}^{2}$$

Here, *k_c_* is the spring constant of pulling device (*e.g.,* AFM), *R* is the distance between two alpha carbon atoms that are stretched, and *u* is the pulling speed. On the other hand, for stretching a molecule with a constant force, the effective potential becomes

(12) $$\tilde{V}=V-\text{F}\cdot \text{R}$$

Here, **F** is the force vector (representing the constant force) and **R** is the vector connecting two alpha carbon atoms that are stretched.

**Figure 6.**
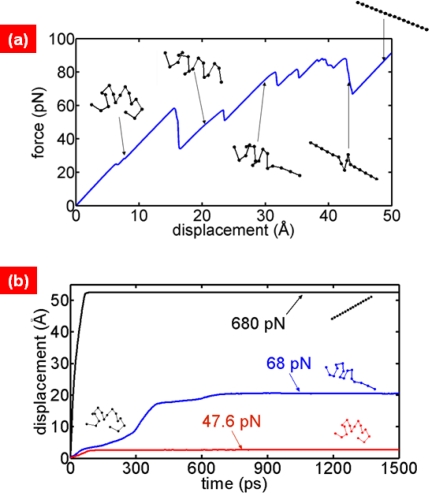
(a) Force-extension curve for alpha helix (pdb: 1akg) obtained from coarse-grained MD simulation using Gō potential field. Here, the simulation considers the stretching of alpha helix with constant pulling speed. Inset shows the carbon backbone structures corresponding to force peaks. It is shown that force peak in saw tooth-like pattern indicates the rupture of hydrogen bonds in alpha helix. (b) Time-extension curve for alpha helix obtained from coarse-grained MD simulation, where alpha helix is stretched with constant force applied to termini. It is shown that, at very small force, the folding structure of alpha helix is unlikely to be unfolded. At high force, the all-or-none fashion of unfolding has been observed for alpha helix.

Figure 6 shows the force-extension curve for alpha helix (pdb code: 1akg) that are extended with constant pulling speed of *u* = 1.67 × 10^–3^ Å/ps. In the force-extension curve, the saw tooth-like patterns are observed as expected. The force peak in the saw tooth-like pattern corresponds to the rupture of hydrogen bonds. The constant-force stretching simulation is also suggested in Figure 6, which shows the time vs. extension.

It is shown that, if the force is very small compared to the thermal energy, then a constant force cannot induce the mechanical unfolding of the alpha helix. For a relatively large force, it is interestingly shown that the hydrogen bonds of the alpha helix are ruptured in an “all or none” fashion. Based on constant-force stretching simulation, the relationship between unfolding time and force can be obtained straightforwardly [83]. Such a relationship can be related to bond rupture models [14,59,66] such as Bell’s model [57] and so forth. This allows one to verify the bond rupture models based on coarse-grained MD simulations of single-molecule pulling experiment [84].

## 5. Statistical Mechanics-Based Models: Coarse-Grained Chain Molecule Models for Protein Unfolding Mechanics

**Figure 7.**
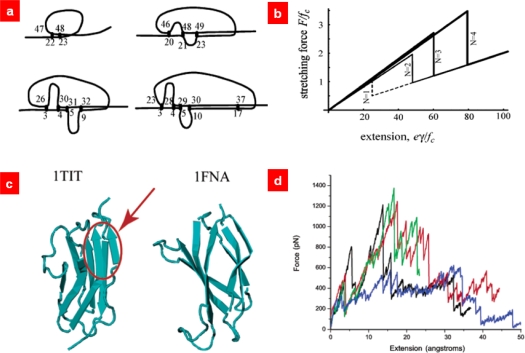
(a) Optimal configuration of cross-links for cross-linked polymer chain molecules from optimization problem. (b) Force-extension curve of such cross-linked polymer chains. The all-or-none fashion of rupture of cross-links has been observed for optical cross-link configurations. (c) Two protein domains such as immunoglobulin (Ig) domain and fibronectin (fn) III domain are considered, since the molecular structure of Ig domain is quite similar to that of fn III domain except the parallel strand, similar to optical cross-link configuration, for Ig domain. (d) Force-extension curves of Ig domain (black, red, and green solid lines) and fn III domain (blue solid line) show that parallel strand enhances the mechanical resistance of protein domain. Figures are adopted from Ref. [85].

As stated above, the WLC model [30,31] suggested in polymer physics is able to describe the entropic elastic behavior of DNA molecules and/or elastic behavior of proteins (before protein unfolding occurs). This indicates that protein unfolding mechanics can be delineated by chain models in statistical mechanics with appropriate an bond rupture model. For instance, Rief *et al*. [40] suggested that a WLC model with Bell’s model for protein domain unfolding is capable of describing protein unfolding behavior quantitatively in a way comparable to that obtained from AFM single-molecule pulling experiments. In order to understand the role of native topology on protein unfolding mechanics, Eom *et al*. [85,86] have considered the cross-linked polymer chain molecule based on a Gaussian chain with formation of cross-links (see Figure 7). It was shown that specific configuration of cross-links similar to parallel strands plays a role on mechanical strength. Kreuzer and coworkers [87,88] have employed statistical mechanics theory for quantitative characterization of polymer chains that are stretched by AFM force spectroscopy. In the similar manner, they have also considered statistical mechanics theory with introduction of a bond rupture model for quantitative understanding of single-molecule pulling experiments of muscle protein titin molecule [89]. Recently, Makarov [90] has suggested the Ising-like model that considers the domain-domain interactions during the stretching of protein molecules. In summary, coarse-grained chain models used in statistical mechanics (in polymer physics) can be employed for quantitative characterization of AFM single-molecule pulling experiments as long as appropriate bond rupture models are considered.

## 6. Micromechanics Model for Mechanical Characterization

**Figure 8.**
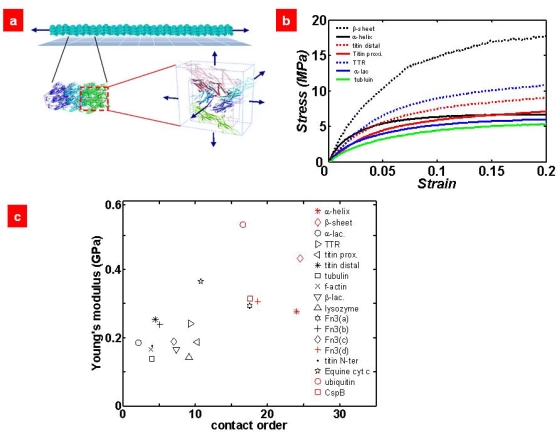
(a) Schematic illustration of micromechanics model of protein materials: It is assumed that representative volume element (RVE) containing the protein crystal with given space group constitutes the protein materials in the repetitative manner. For mechanical characterization, RVE is stretched according to application of constant, normal strain field to RVE. Based on virial stress theory, the stress-strain relation is obtained so as to provide the elastic modulus of protein materials. (b) Stress-strain curves for various protein materials with assumption of Gō-like potential prescribed to protein crystal in RVE. (c) Relationship between contact order (CO) and Young’s modulus of protein materials shows the role of native topology on the elastic properties of protein materials. Figures are adopted from Ref. [93].

Although the mechanical response of proteins at a single-molecule level provides the detailed mechanism of protein unfolding mechanics, the macroscopic mechanical properties such as Young’s modulus for biological protein materials cannot be quantitatively delineated by single-molecule pulling experiments and/or simulations. Recently, the micromechanics model has been revisited in such a way that the representative volume element (RVE) that contains a protein crystal with a given space group is stretched by constant, discrete, normal strains in order to find the stress acting on the RVE (see Figure 8).

Buehler [91,92] have taken into account the micromechanics model base on all-atom simulations, while Eom and coworkers [93] have considered the coarse-grained model such as the Gō model with micromechanics models. Specifically, the RVE is stretched based on a constant, normal strain, and then the atomic coordinates of the alpha carbon atoms are updated. Since the atomic coordinates updated after application of strain field to RVE is not in an equilibrium state, the equilibrium coordinates of alpha carbon atoms are obtained from energy minimization. At equilibrium state, the virial stress theory [94] was used to compute the atomic stress acting on RVE due to strain field. Once the stress-strain field is found, the Young’s modulus is easily obtained as *E* = ∂*s*/∂*e*, where *e* is the normal strain and *s* is the virial stress. Figure 8 shows the stress-strain relation for given protein materials with assumption of Gō-like model for protein crystal in RVE. Remarkably, it is shown that the contact-order plays a critical role on Young’s modulus as well as yield stress in the protein materials. This sheds light on the role of native topology on the mechanical properties of protein materials.

## 7. Cantilever Bioassay for *in Vitro* Molecular Recognitions

The AFM cantilever-based bioassay has enabled not only the description of the mechanical responses of protein molecules but also a quantitative understanding of molecular interactions related to *in vitro* molecular recognition relevant to early diagnosis of specific diseases [5]. Specifically, the cantilever surface is chemically modified in such a way that receptor molecules are immobilized on the cantilever surface in order to capture the specific target molecules. The detection principle is the direct transduction of molecular interaction on the cantilever surface into the mechanical response change such as cantilever’s bending deflection change and/or resonant frequency shift.

**Figure 9.**
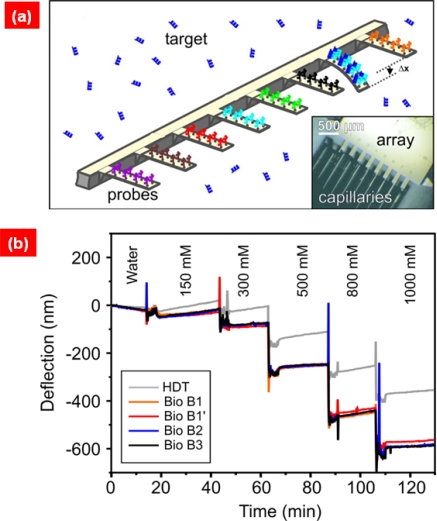
(a) Schematic illustration of functionalized cantilever array for detection of specific biological molecules. Inset shows the microscope image of cantilever array. (b) Cantilever bending deflection change of cantilever array induced by change of ionic strength in the buffer solution. Figures are adopted from Ref. [97].

Gerber and coworkers [28] have studied the intermolecular interactions based on cantilever bioassays. Specifically, the intermolecular interactions generated during the molecular adsorption of alkanethiol chains were studied based on the measurement of surface stress [95] related to cantilever’s bending deflection change arising from such adsorption. In a similar manner, Gerber and coworkers [96,97] have reported the label-free detection of specific DNA molecule by using a cantilever functionalized with single-stranded DNA molecules (see Figure 9). In a similar spirit, Majumdar and coworkers [98] have described the cantilever bioassay-based detection of DNA hybridization, and also studied the role of ionic strength on DNA hybridization. Moreover, they have also suggested the sensitive detection of marker proteins related to specific diseases using a cantilever-based bioassay by measuring the deflection change induced by antigen-antibody binding on a cantilever’s surface [99]. Recently, McKendry and coworkers [100] have studied the resistance of superbugs to drug molecules based on a cantilever bioassay by evaluating the deflection change driven by the interactions between drug molecules and superbugs on the cantilever’s surface. Furthermore, Gerber and coworkers [101] have shown the potential of cantilever bioassays for quantitative characterization of ligand binding onto membrane proteins. These epitomes shed light on cantilever bioassays for not only the understanding of the intermolecular interactions, but also sensitive label-free detection relevant to early diagnosis.

Although cantilever bioassay based on measurement of bending deflection change induced by molecular interactions enables the sensitive label-free detection related to early diagnosis, it does not allow one to gain insight into how many molecules are involved in such interactions [102]. Instead of measurement of bending deflection change, the cantilever bioassay based on evaluation of a cantilever’s resonant frequency shift in response to molecular binding on its surface has been suggested in order to estimate the mass of bound molecules onto a biologically functionalized cantilever.

Specifically, the resonant frequency shift is linearly proportional to the total mass of adsorbed molecules [103], as long as the thickness of adsorbed molecular layer is much smaller than the cantilever’s thickness [104]. Moreover, the smaller the cantilever is, the smaller the amount of molecules whose label-free detection is possible with the cantilever. Roukes and coworkers [105] have reported the highly sensitive detection of chemical molecules even at zepto-gram resolution equivalent to atomic mass using NEMS resonators. Moreover, they have also used nanomechanical mass spectrometry to measure the molecular weight of a single protein using NEMS resonators [106].

**Figure 10.**
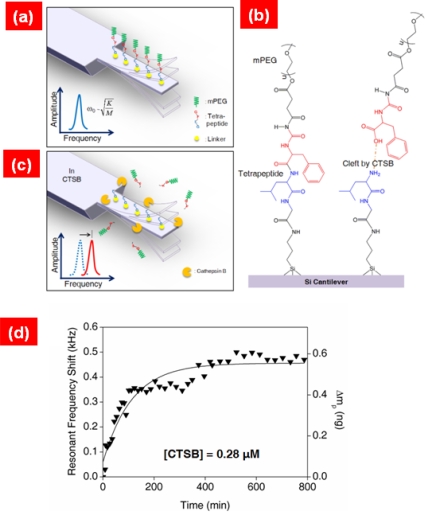
Schematic illustration of *in situ* monitoring of proteolysis of peptide by protease based on resonant microcantilever immersed in buffer solution: (a) A cantilever functionalized by peptide chains is vibrating with the resonance of *ω*_0_. (b) Chemical structure of peptide chain and/or cleft peptide chain by protease. (c) Enzymatic cleavage of peptide due to protease increases the resonance of microcantilever due to decrease of molecular mass driven by proteolysis. (d) Resonant frequency shift due to enzymatic cleavage of peptide is well described by Langmuir kinetics. Figures are adopted from Ref. [110].

Despite their capability of highly sensitive detection at atomic resolution, NEMS resonators are inappropriate for label-free detection of biological molecules in liquid environments because of their low quality factor (Q-factor) in liquids [107]. *In situ*, real-time detection of biomolecular interactions in liquid is essential for gaining insight into the kinetics of biomolecular interactions. Recently, Kwon *et al*. [108,109] have reported the *in situ*, real-time detection of CRP (C reactive protein) antigen-antibody interactions [108] as well as DNA hybridization [109] using resonant microcantilevers that exhibit the relatively high quality factor in buffer solution. It is remarkably shown that a resonant frequency shift, which was measured in buffer solution using resonant microcantilever, due to biomolecular interactions has been well depicted by Langmuir kinetic model [109]. Moreover, Kwon *et al*. [110] have described the label-free, *in situ*, real-time detection of proteolysis of tetrapeptide sequences driven by enzymatic activity by using a resonant microcantilever immersed in buffer solution (see Figure 10).

It is interestingly shown that the kinetic rate of proteolysis of a tetrapeptide by protease can be extracted from the resonant frequency shift induced by enzymatic cleavage of the tetrapeptide. It is implied that a resonant microcantilever immersed in a fluid may enable one to quantitatively understand the kinetics of various molecular interactions such as protein-protein interactions, protein-DNA interactions, protein-enzyme interaction, and/or protein-drug interactions.

For highly sensitive, *in situ* detection of specific molecules in fluids, Manalis and coworkers [111,112] have introduced the suspended microchannel resonator (SMR), that is, a resonant microcantilever in which a microchannel is embedded. Since molecular detection occurs inside the microchannel embedded in the microcantilever, SMR exhibits a high Q-factor during the *in situ* detection of biomolecules in fluids. This enables the highly sensitive, label-free detection of specific molecules. It is shown that SMR allows the measurement of total mass of target biomolecules and/or a cell captured by probe molecules immobilized in the microchannel [112]. Moreover, it is also found that the resonance behavior of SMR is dependent on the viscosity of fluid inside the microchannel, implying the ability of SMR to measure the viscosity of fluids [113]. However, development of a small-scale SMR remains a challenge, however, because of the difficulties associated with fabrication of nanochannels inside the small-scale cantilever.

## 8. Conclusions

In this article, we have described the current state-of-art in single-molecule mechanics bioassay based on AFM bioassays and/or LT bioassays. Single-molecule pulling experiments using AFM bioassays and/or LT bioassays have enabled the quantitative characterization of the mechanical responses of single biological molecules such as proteins, DNA, and/or RNA, in order to understand their mechanical functions. More remarkably, single-molecule pulling experiments have also allowed the verification of statistical mechanics theories such as Jarzynski’s theorem and/or Crooks’ theorem based on unfolding and/or refolding process of biomolecules. Moreover, single-molecule pulling experiments have validated bond rupture models, which relate the kinetic rate of bond rupture to mechanical force. This indicates that single-molecule pulling experiments can provide a quantitative understanding of protein unfolding and/or folding processes, which play a significant role in biological functions and/or malfunctions.

Although single-molecule pulling experiments provide a quantitative characterization of protein unfolding mechanics, such experiments cannot suggest the detailed mechanisms involved, such as unfolding pathways. As stated above, coarse-grained MD simulations have enabled the quantitative understanding of protein unfolding mechanism with details of molecular structural deformation by mechanical force. Moreover, the coarse-grained chain models have been briefly introduced for quantitative understanding of elastic response of proteins obtained from single-molecule pulling experiments. In addition, the micromechanics models with coarse-grained MD model and/or MD simulations provide insight into the macroscopic mechanical properties, such as Young’s modulus, of biological protein materials. This highlights the use of coarse-grained MD simulations and/or coarse-grained chain models for quantitative insight into detailed mechanisms of mechanical responses of biological liquid crystals.

Moreover, AFM bioassays can be employed not only for single-molecule pulling experiments (that provide the mechanical responses of biomolecules) but also label-free detection related to early diagnosis of specific diseases. The detection principle is the transduction of molecular interactions into the cantilever’s bending deflection change (static mode) and/or resonant frequency shift (dynamic mode). We have reviewed the current state-of-art in label-free detection using both static-mode and dynamic-mode cantilevers. It is suggested that cantilever-based bioassays enable the quantitative characterization of various biomolecular interactions such that their kinetics.

In summary, we have discussed AFM-based single-molecule bioassays from single-molecule pulling experiments to cantilever-based label-free detection. This implies that AFM bioassays are one of the rudimentary tools available for quantitative understanding of not only the mechanical responses of a single biomolecule but also biomolecular interactions (and their kinetics). AFM bioassays with computational simulations can also provide insight into the biological functions of biological molecules and/or biomolecular interactions.
